# Bone Marrow Absorbed Doses and Correlations with Hematologic Response During ^177^Lu-DOTATATE Treatments Are Influenced by Image-Based Dosimetry Method and Presence of Skeletal Metastases

**DOI:** 10.2967/jnumed.118.225235

**Published:** 2019-10

**Authors:** Linn Hagmarker, Johanna Svensson, Tobias Rydén, Martijn van Essen, Anna Sundlöv, Katarina Sjögreen Gleisner, Peter Gjertsson, Peter Bernhardt

**Affiliations:** 1 Department of Radiation Physics, Sahlgrenska Academy, University of Gothenburg, Gothenburg, Sweden; 2 Department of Oncology, Sahlgrenska Academy, University of Gothenburg, Gothenburg, Sweden; 3 Department of Clinical Physiology, Sahlgrenska University Hospital, Gothenburg, Sweden; 4 Department of Oncology, Skåne University Hospital, Lund, Sweden; 5 Lund University, Division of Oncology and Pathology, Department of Clinical Sciences, Lund, Sweden; and; 6 Department of Medical Radiation Physics, University of Lund, Lund, Sweden

**Keywords:** bone marrow dosimetry, hematologic response, ^177^Lu-DOTATATE

## Abstract

This study aimed to compare different image-based methods for bone marrow dosimetry and study the dose–response relationship during treatment with ^177^Lu-DOTATATE in patients with and without skeletal metastases. **Methods:** This study included 46 patients with advanced neuroendocrine tumors treated with at least 2 fractions of ^177^Lu-DOTATATE at Sahlgrenska University Hospital. High- and low-uptake compartments were automatically outlined in planar images collected at 2, 24, 48, and 168 h after injection. The bone marrow absorbed doses were calculated from the cross doses of the high- and low-uptake compartments and the self-dose, using the time–activity concentration curve for the low-uptake compartment. This time–activity concentration curve was adjusted using a fixed constant of 1.8 for the planar dosimetry method and using the activity concentrations in vertebral bodies in SPECT images at 24 h after injection of ^177^Lu-DOTATATE in 4 hybrid methods: L4-SPECT used the activity concentration in the L4 vertebra, whereas V-SPECT, L-SPECT, and T-SPECT used the median activity concentration in all visible vertebrae, lumbar vertebrae, and thoracic vertebrae, respectively. **Results:** Using the planar method, L4-SPECT, V-SPECT, L-SPECT, and T-SPECT, the estimated median bone marrow absorbed doses were 0.19, 0.36, 0.40, 0.39, and 0.46 Gy/7.4 GBq, respectively, with respective ranges of 0.12–0.33, 0.15–1.44, 0.19–1.71, 0.21–1.60, and 0.18–2.12 Gy/7.4 GBq. For all methods, the bone marrow absorbed dose significantly correlated with decreased platelet counts. This correlation increased after treatment fraction 2: the Spearman correlation (*r*_s_) were −0.49 for the planar method, −0.61 for L4-SPECT, −0.63 for V-SPECT, −0.63 for L-SPECT, and −0.57 for T-SPECT. A separate analysis revealed an increased correlation for patients without skeletal metastases using the planar method (*r*_s_ = −0.67). In contrast, hybrid methods had poor correlations for patients without metastases and stronger correlations for patients with skeletal metastases (*r*_s_ = −0.61 to −0.74). The mean bone marrow absorbed doses were 3%–69% higher for patients with skeletal metastases than for patients without. **Conclusion:** The estimated bone marrow absorbed doses by image-based techniques and the correlation with platelets are influenced by the choice of measured vertebrae and the presence of skeletal metastases.

Peptide receptor radionuclide therapy with ^177^Lu-DOTATATE is a valuable treatment option for metastatic neuroendocrine tumors that positively impacts survival parameters and has only minor side effects (1,2). However, treatment is restricted because of limitations imposed by irradiation of the kidneys and bone marrow. Current evidence indicates that the kidneys can tolerate mean absorbed doses above the general dose limit of 23 Gy and that patients can receive more than the standard of 4 fractions of 7.4 GBq (3–5). Recent studies have also reported the use of retreatment for patients with progressed disease (6,7). If higher renal mean absorbed doses are accepted, higher total activity will be administered and bone marrow toxicity might become the dose-limiting factor.

A dose limit of 2 Gy to the bone marrow is based on treatments with ^131^I and blood-based dosimetry (8,9). In a recently published study, blood-based dosimetry was performed for ^177^Lu-DOTATATE treatments in 200 patients and showed no correlation with hematologic toxicity (10). Bergsma et al. has presented the only correlation for ^177^Lu-DOTATATE using blood-based dosimetry for a small selected subpopulation (11). It remains unclear if this method is valid for bone marrow dosimetry for ^177^Lu-DOTATATE (12).

Image-based bone marrow dosimetry and its correlation with toxicity is challenging because of problems quantifying the activity concentrations in small dispersed bone marrow cavities, which may be infiltrated with metastases. Additionally, the bone marrow is mixed with adipose tissue, with a fraction that differs between vertebrae and with age and sex (13–15). Moreover, toxicity is dependent on the bone marrow status, which can be affected by several factors such as age and previous treatments. Although most patients receiving peptide receptor radionuclide therapy experience minor side effects, 10% develop severe hematologic toxicity and 1%–2% develop myelodysplastic syndrome and acute leukemia (16–20). Personalized bone marrow dosimetry should be included to prevent bone marrow toxicity during treatment and the risk of myelodysplastic syndrome and acute leukemia (16). However, the significance of image-based bone marrow dosimetry as a predicting factor for bone marrow toxicity is unclear.

Our research group previously published a planar image–based method for bone marrow dosimetry showing a statistically significant correlation between the bone marrow absorbed dose and hematologic toxicity (21). The accuracy of this method may be reduced because it uses a fixed ratio between the activity concentration in bone marrow and in low-uptake organs, without accounting for individual ratios. Moreover, the method does not account for cross irradiation from infiltrating metastases and thus may underestimate the bone marrow absorbed dose in patients with skeletal metastases.

Here, we aimed to further develop this methodology into a hybrid planar and SPECT image method, enabling more personalized dosimetry. Because the active bone marrow varies within vertebrae, we determined the activity concentration in vertebrae using several methods. We also investigated whether the bone marrow absorbed dose correlates with hematologic response early during treatment. Finally, we investigated if skeletal metastases influence the dose–response relationship, by dividing patients into 3 groups: all patients, patients without skeletal metastases, and patients with skeletal metastases.

## MATERIALS AND METHODS

The patients were included in the ILUMINET study (EUDRACT no. 2011‐000240‐16), which is a collaboration between Sahlgrenska University Hospital Gothenburg and Skåne University Hospital, Lund, Sweden. This prospective study was approved by the regional Ethics Review Board in Gothenburg and was performed in accordance with the Declaration of Helsinki and national regulations.

### Patients and Therapy

This cohort study included 46 patients diagnosed with advanced neuroendocrine tumors treated with at least 2 fractions of ^177^Lu-DOTATATE at Sahlgrenska University Hospital between 2011 and 2017 (Table 1). The mean administered activity for this cohort was 7.5 GBq (range, 6.8–8.0 GBq) of ^177^Lu-DOTATATE per treatment fraction. Fractions were administered approximately 8 wk apart until reaching a mean renal biologic effective dose of 27 ± 2 Gy or until the patient exhibited a persisting hematologic response or disease progression. Each fraction was coadministered with an intravenous infusion of kidney-protective amino acids (2.5% lysine and 2.5% arginine in 1 L of 0.9% NaCl; infusion rate, 250 mL/h). Infusions of ^177^Lu-DOTATATE and amino acids were administered over 30 min and 4 h, respectively. Weekly blood samples were drawn to detect treatment-related toxicity, and the relative nadirs of the number of platelet counts versus baseline were used to evaluate dose–response relationships.

**TABLE 1:** Patient Characteristics

Characteristic	Data
Sex	
Female	22 (47.8)
Male	24 (52.2)
Age	
All patients	64 (35–84)
Female	63.5 (44–84)
Male	64 (35–78)
Primary tumor	
Small intestine	28 (60.9)
Pancreas	7 (15.2)
Lung	3 (6.5)
Colorectal	2 (4.3)
Other/unknown	6 (13.0)
Ki-67 index	
0%–2%	21 (46.7)
3%–20%	25 (53.3)
>20%	0
Skeletal metastases	
All patients	24 (52.2)
Female	9 (40.9)
Male	15 (62.5)
Baseline platelets (10^9^/L)	
All patients	241 (128–519)
Female	250.5 (128–503)
Male	264 (150–519)
Previous treatments	
Somatostatin analogs	33 (71.7)
Surgery	42 (91.3)
Everolimus or sunitinib	5 (10.9)
Chemotherapy	11 (23.9)
Locoregional therapy	30 (65.2)
PRRT	5 (10.9)
^ 131^I-MIBG*	1 (2.2)
Performance status (ECOG)	
0	28 (60.0)
1	17 (37.8)
2	1 (2.2)
3–4	0

* Metaiodobenzylguanidine.

ECOG = Eastern Cooperative Oncology Group.

Qualitative data are expressed as numbers followed by percentages in parentheses; continuous data are expressed as mean ± SD.

### Image Acquisition

During each treatment fraction, we acquired 4 planar images, at 2, 24, 48, and 168 h after injection, and a SPECT/CT scan at 24 h after injection. Imaging was performed using a Tandem Discovery Pro, Tandem Discovery, Infinia, or Millennium VG (GE Healthcare) and a Picker IRIX (Phillips). Planar whole-body scintigraphy (anterior and posterior) was performed with a scanning time of 10 cm/min. The camera was equipped with a medium-energy general-purpose collimator, and the energy window was set at 208.4 keV  ±  10%. No scatter correction was applied. Using the same energy settings, SPECT imaging was performed with a 30-s frame duration for 120 projections. SPECT reconstructions were completed using the Monte Carlo–based reconstruction code SARec in the image platform PhONSAi (22). Scintigraphy of a Petri dish containing ^177^Lu placed at different depths in a tissue-equivalent phantom was used to determine the sensitivity and effective attenuation coefficient of the γ-cameras.

### The Planar 2-Compartment Method

**FIGURE 1.**
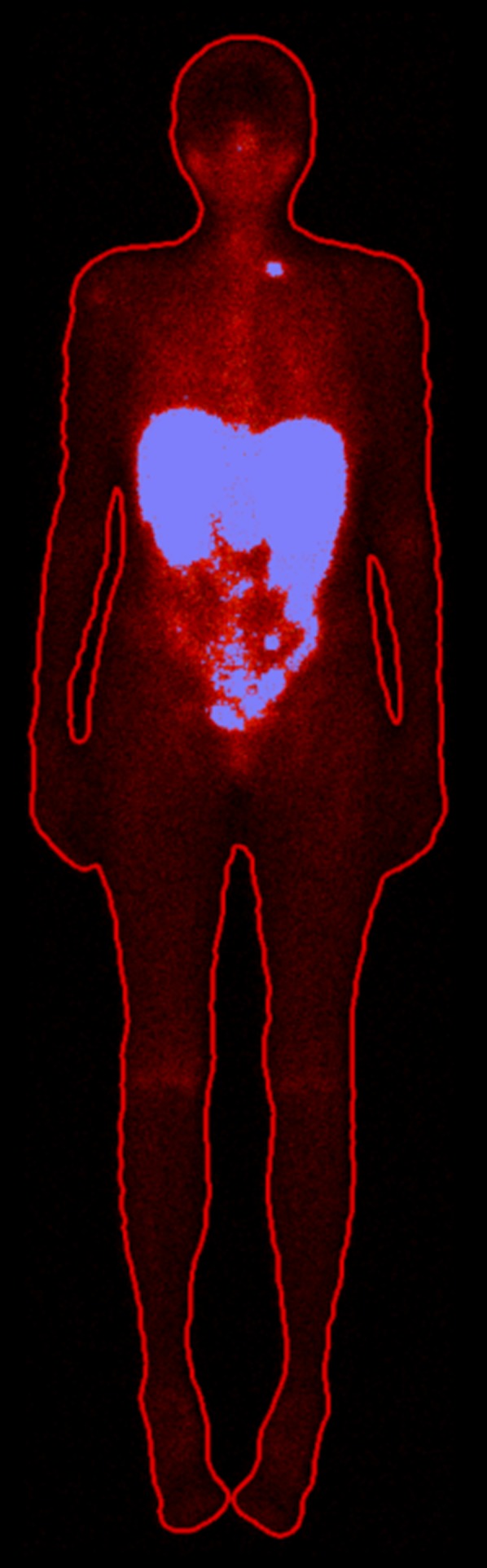
Resulting segmentation of 2 compartments in planar image. High-uptake compartment (blue) comprises liver, spleen, kidneys, and tumors, and low-uptake compartment comprises rest of body.

For each patient, the whole body was divided into a high-uptake and a low-uptake compartment by creating a whole-body region of interest in filtered geometric mean images produced from the acquired anterior and posterior planar images. This whole-body region of interest was applied to unfiltered images, and an automated threshold-based segmentation tool in the image platform PhONSAi was used to segment the whole body into the 2 compartments (Fig. 1) (21,23). The algorithm uses the optimal threshold for segmentation, according to previous work (21). Segmentation produced a high-uptake compartment comprising the liver, spleen, kidneys, and tumor and a low-uptake compartment comprising the rest of the body.

### Dose Calculation: The Planar 2-Compartment Method

The bone marrow absorbed dose was calculated as the sum of the self-dose from the bone marrow itself plus the cross doses from the high- and low-uptake compartments (Eq. 1). Activity in the planar images was quantified using the conjugate view formula, using the body thickness measured over the abdomen from CT images and a general organ thickness of 8 cm (21). Time–activity curves were obtained using the activities quantified from the planar images. For the low-uptake compartment, we used a biexponential fit. For the high-uptake compartment, we used a linear fit between the first 2 time points and an exponential fit between the second and fourth. The time–activity concentration curve for bone marrow was created by dividing the activity in the low-uptake compartment by the mass of the low-uptake compartment. In a previous work, a ratio of 1.8 was found between the SPECT-derived activity concentration in the bone marrow in vertebra L4 and the activity concentration in the surrounding tissue, which was assumed to represent the low-uptake compartment (23). This ratio was used for each patient.

From the time–activity curves, we determined the time-integrated activity. This was used to calculate the bone marrow absorbed dose ($D_{BM})$ together with the S factors for each compartment, according to Equation 1:

Eq. 1 $$D_{\text{BM}}={\tilde{C}}_{\text{BM}}\times \phi_{\text{BM}\leftarrow \text{BM}}\times \Delta \times 1.8+{\tilde{A}}_{\text{low}}\times S_{\text{BM}\leftarrow low}+{\tilde{A}}_{\text{high}}\times S_{\text{BM}\leftarrow \text{high}}.$$

In this equation, ${\tilde{C}}_{\text{BM}}$ is the time-integrated activity concentration in bone marrow; $\phi_{\text{BM}\leftarrow \text{BM}}$ is the absorbed fraction for self-irradiation, which was set to 1; Δ is the energy released per disintegration, which was set to 147 keV (24); ${\tilde{A}}_{\text{low}}$ and ${\tilde{A}}_{\text{high}}$ are the time-integrated activities in the low- and high-uptake compartments, respectively; and $S_{\text{BM}\leftarrow \text{low}}\;$and $S_{\text{BM}\leftarrow \text{high}}\;$are the cross-dose S factors calculated using specific absorbed fractions for all emitted γ-energies for adult men and women (25) and weighted on the basis of the masses of the organs included in the low- and high-uptake compartments, respectively, using previously reported average organ masses for men and women. The mean absorbed doses were estimated for treatment fractions 1 and 2.

### The Hybrid Methods

**FIGURE 2.**
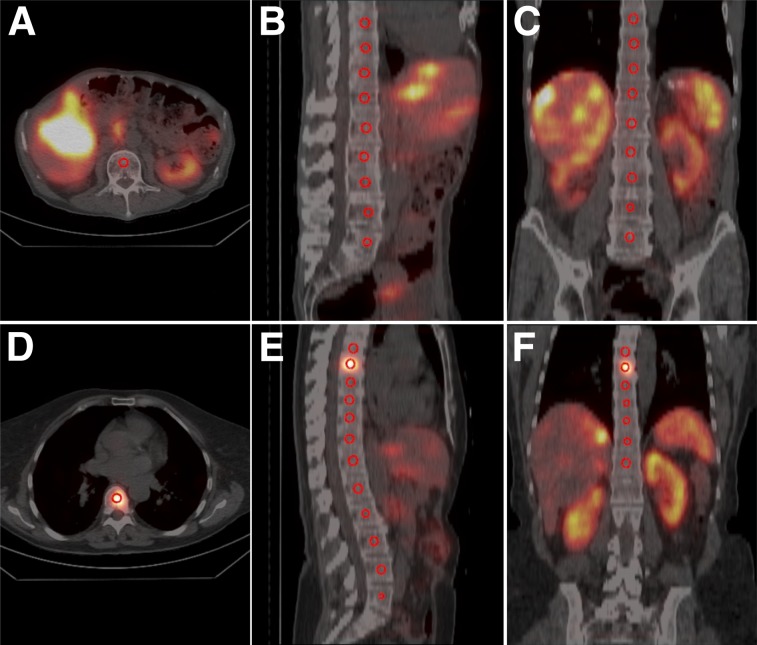
Location of spheric volume of interests in vertebrae. (A–C) Volumes of interest in SPECT/CT images of patient without skeletal metastases. (D–F) Spheric volumes of interest in SPECT/CT images of patient with skeletal metastasis in thoracic vertebra 7.

In CT images, a spheric volume of interest was created in the middle of the body of each visible vertebra (Figs. 2A–2C), representing the bone marrow. To reduce partial-volume effects and minimize cross contamination from surrounding high-uptake regions, the spheres were smaller than the vertebrae, having a volume of 0.7 cm^3^, and were centrally placed within the vertebrae. These spheres were then transferred to the reconstructed SPECT image, and the activity concentration was calculated within the spheres for each patient. To generate a time–activity concentration curve for the bone marrow, we divided the activity in the low-uptake compartment by the mass of the low-uptake compartment and adjusted the curve using the activity concentration in the spheres. The mass of the low-uptake compartment was determined as the patient-specific weight minus the mass of the high-uptake compartment, which was calculated as the area of the high-uptake compartment multiplied by the abdominal thickness. A density of unity was assumed for the 2 compartments. We investigated 4 variations of this hybrid method. The L4-SPECT method used the activity concentration in L4 (L5 if L4 was infiltrated with metastasis and thereafter L3 and L2). The V-SPECT, L-SPECT, and T-SPECT methods used the median value of the activity concentration in all visible vertebrae, lumbar vertebrae, and thoracic vertebrae, respectively.

We examined the dose response for 3 patient groups: all patients, patients without skeletal metastases (Figs. 2A–2C), and patients with skeletal metastases (Figs. 2D–2E). We also determined the activity distribution in the vertebrae in patients without skeletal metastases. Because of the reported difference in adipose tissue between women and men in L1–L4, we determined the mean activity concentrations in these vertebrae for women and men (14,15).

### Absorbed Dose Calculation: The Hybrid Methods

The bone marrow absorbed dose for the hybrid methods ($D_{\text{BM,SPECT}})$ was calculated according to Equation 2. The planar method used a general ratio between the low-uptake compartment and the bone marrow. In contrast, here the time–activity concentration curve created for the low-uptake compartment was adjusted using the activity concentration determined from the spheres in the SPECT/CT image for each patient.

Eq. 2 $$D_{\text{BM,SPECT}}={\tilde{C}}_{\text{BM,SPECT}}\times \phi_{\text{BM}\leftarrow \text{BM}}\times \Delta +{\tilde{A}}_{\text{low}}\times S_{\text{BM}\leftarrow \text{low}}+{\tilde{A}}_{\text{high}}\times S_{\text{BM}\leftarrow \text{high}}.$$

In this equation, ${\tilde{C}}_{\text{BM,SPECT}}$ is the time-integrated activity concentration calculated from the adjusted time–activity curve for the low-uptake compartment. The other parameters have been described for Equation 1. The absorbed doses were estimated for treatment fractions 1 and 2.

### Statistical Analysis

The dose–response relationships were examined by Spearman correlation (*r*_s_). *P* values of less than 0.05 were considered significant. The comparison of the absorbed doses estimated with the planar and hybrid methods, the comparison of absorbed doses between patients with and without skeletal metastases, and the comparison of activity concentrations in the lumbar vertebrae in men and women were performed using the Wilcoxon rank sum test.

## RESULTS

### Bone Marrow Absorbed Dose

**FIGURE 3.**
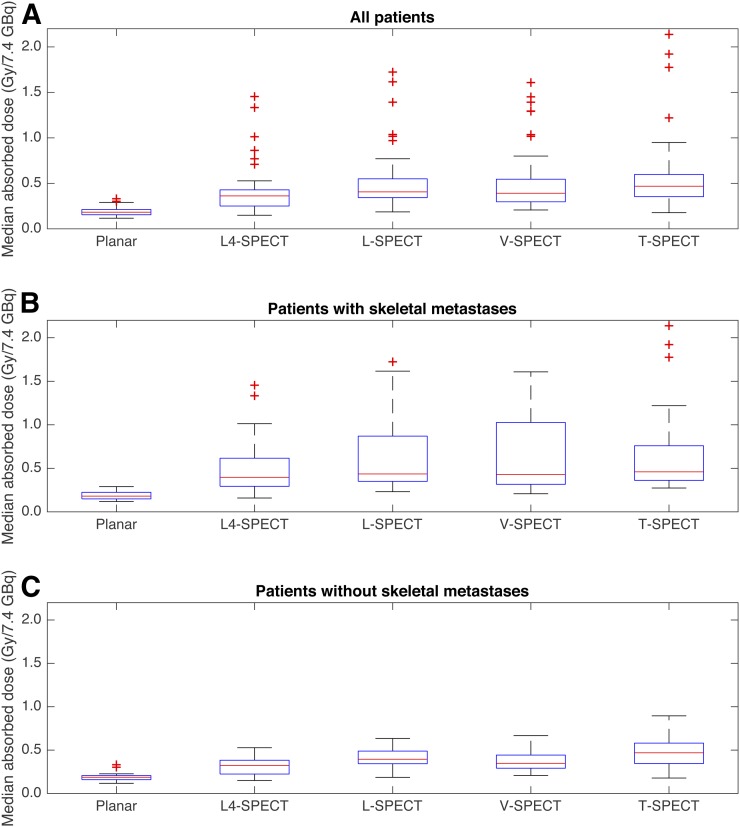
Median bone marrow absorbed doses after treatment fraction 1 estimated using planar method and 4 hybrid methods for all patients (A), patients with skeletal metastases (B), and patients without skeletal metastases (C).

In the first treatment fraction using the planar method and L4-SPECT, V-SPECT, L-SPECT, and T-SPECT, the median absorbed doses for patients without skeletal metastases were 0.19, 0.32, 0.39, 0.34, and 0.46 Gy/7.4 GBq, respectively, with respective ranges of 0.12–0.32, 0.15–0.5, 0.18–0.62, 0.21–0.67, and 0.18–0.86 Gy/7.4 GBq (Fig. 3). For patients with skeletal metastases, the corresponding median absorbed doses were 0.18, 0.39, 0.44, 0.42, and 0.45 Gy/7.4 GBq, respectively, with respective ranges of 0.12–0.29, 0.16–1.4, 0.23–1.7, 0.21–1.6, and 0.27–2.1 Gy/7.4 GBq. The results show that the planar method had a similar range of absorbed doses for patients with or without metastases. In contrast, when skeletal metastases were present, the hybrid methods had a higher range in absorbed doses and the median absorbed dose was increased by 13%–22% (L4-SPECT, V-SPECT, and L-SPECT).

The absorbed doses estimated using the hybrid methods were significantly higher than the absorbed dose estimated using the planar method for the whole patient cohort, the patients with skeletal metastases, and the patients without skeletal metastases. Absorbed doses estimated using L4-SPECT were significantly higher for patients with skeletal metastases than for patients without metastases.

When we studied the activity distribution among vertebrae in the 22 patients who did not have skeletal metastases, the highest activity concentration was between T10 and L1, whereas the lowest values were for T5*–*T7 and L4–L5 (Table 2). This finding also reflects the tendency for higher absorbed doses using T-SPECT, which mainly uses the median of T8–T12. The 22 patients were also grouped according to sex. The mean activity concentration in L1–L4 was 1.13 times higher in men, but this difference was not statistically significant.

**TABLE 2:** Median Activity Concentration in Each Vertebra Among 22 Patients Without Skeletal Metastases

Vertebra	Median activity concentration and range (kBq/mL)	Patients (*n*)
T5	14.4	1
T6	20.2 (8.7–27.7)	3
T7	21.3 (10.5–37.9)	5
T8	25.8 (6.6–41.1)	9
T9	28.1 (14.6–64.6)	15
T10	37.1 (14.8–86.0)	21
T11	47.8 (10.4–77.4)	22
T12	41.0 (16.1–70.5)	22
L1	38.4 (15.1–79.2)	22
L2	35.6 (19.0–81.4)	22
L3	28.2 (11.7–56.3)	22
L4	23.9 (11.7–46.0)	22
L5	24.2 (7.7–51.4)	21

### Dose–Response Relationship

**FIGURE 4.**
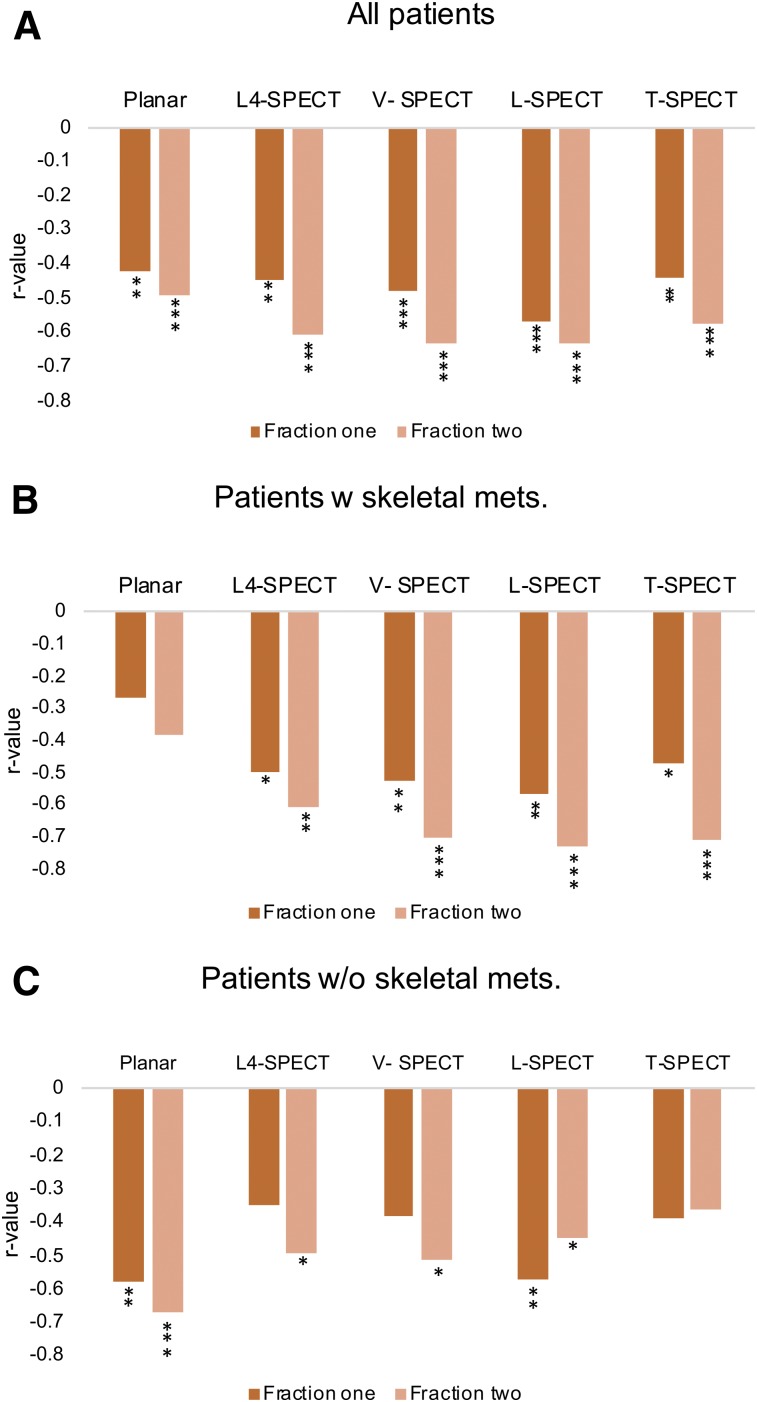
*r* values for dose–response relationships between bone marrow absorbed dose and decrease in platelet counts when using planar method and hybrid methods for treatment fractions 1 and 2. (A) *r* values when all patients are included. (B) *r* values for patients with skeletal metastases. (C) *r* values for patients without skeletal metastases. *P < 0.05. **P < 0.01. ***P < 0.001.

**FIGURE 5.**
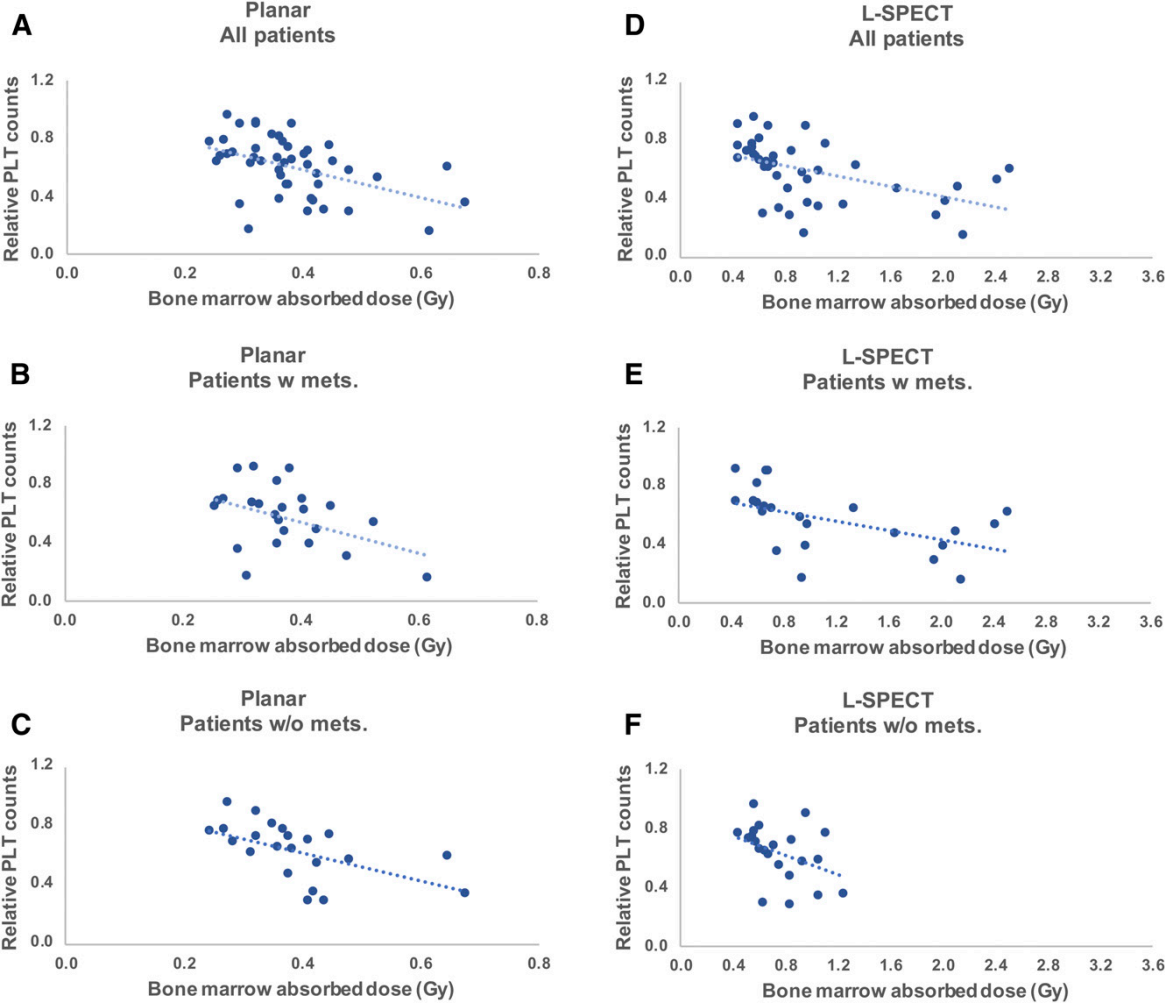
Total bone marrow absorbed doses vs. response of platelet (PLT) counts after 2 treatment fractions using planar method (A–C) and hybrid method L-SPECT (D–F). Patients are divided into 3 groups: all patients, patients with skeletal metastasis, and patients without skeletal metastasis. Dotted lines represent linear regressions, for illustrative purposes.

When including all patients, significant dose–response relationships were established after the first treatment fraction between the bone marrow absorbed dose and the relative decrease in platelet counts using the planar method (*r*_s_ = −0.42, *P* < 0.001), L4-SPECT (*r*_s_ = −0.45, *P* < 0.01), V-SPECT (*r*_s_ = −0.48, *P* < 0.001), L-SPECT (*r*_s_ = −0.57, *P* < 0.0001), and T-SPECT (*r*_s_ = −0.44, *P* < 0.01) (Figs. 4 and 5). The correlations increased for fraction 2 using the cumulative absorbed dose, at an *r*_s_ of −0.49 for the planar method (*P* < 0.001), −0.61 for L4-SPECT (*P* < 0.0001), −0.63 for V-SPECT (*P* < 0.0001), −0.63 for L-SPECT (*P*_2_ < 0.0001), and −0.57 for T-SPECT (*P* < 0.0001).

We then performed a separate analysis of patients with and without skeletal metastases. For patients without skeletal metastases, the planar method showed stronger significant dose–response relationships after treatment fractions 1 and 2 (*r*_s_ = −0.58 and *r*_s_ = −0.67, respectively) (Fig. 4C), whereas no statistically significant correlation was established using the planar method for patients with skeletal metastases (Fig. 4B). Using the hybrid methods, a significant correlation could be established in patients without skeletal metastases using L-SPECT (*r*_s_ = −0.57) in treatment fraction 1 and using L4-SPECT (*r*_s_ = −0.49), V-SPECT (*r*_s_ = −0.51), and L-SPECT (*r*_s_ = −0.45) in treatment fraction 2. For patients with skeletal metastases, significant dose–response relationships were established after treatment fractions 1 and 2 using L4-SPECT (*r*_s_ = −0.50 and *r*_s_ = −0.61, respectively), V-SPECT (*r*_s_ = −0.53 and *r*_s_ = −0.70, respectively), L-SPECT (*r*_s_ = −0.57 and *r*_s_ = −0.74, respectively), and T-SPECT (*r*_s_ = −0.48 and *r*_s_ = −0.71, respectively) (Fig. 4B). All dosimetry and response data are available as supplemental tables (supplemental materials are available at http://jnm.snmjournals.org).

## DISCUSSION

The present study aimed to update a bone marrow dosimetry method based on planar images into a hybrid methodology that incorporates SPECT/CT for patient-specific determination of activity in bone marrow. We expected this would lead to an improved correlation between the bone marrow absorbed dose and platelet response. However, this improvement was only partly achieved. When the planar method was used, the predictive ability showed a modest correlation after the first fraction and a stronger correlation for fraction 2. There was also a strikingly better response prediction for patients without metastases than with metastases. This finding suggested that the planar methodology is suitable for patients without skeletal metastases but less so for patients with metastases. The same should be true for blood-based dosimetry. In a recent prospective study including 200 patients with neuroendocrine tumors treated with ^177^Lu-DOTATATE, 22% of patients had to stop treatment because of bone-marrow–related events even though none had reached the 2-Gy dose limit (10). The authors used blood-based dosimetry, which showed low mean specific absorbed doses (0.12 Gy/7.4 GBq), and concluded that the dosimetry method did not predict toxicity. This might reflect the inability of a blood-based methodology to take into account the impact of infiltrating skeletal metastases and to correctly estimate the individual variations in bone marrow activity concentration.

The planar method yielded a median bone marrow absorbed dose of 0.18 Gy/7.4 GBq, whereas the hybrid methods yielded absorbed doses between 0.32 and 0.46 Gy/7.4 GBq, which are comparable to other published bone marrow dosimetry data (3,10,11,26–29). The low absorbed doses for the planar method are due to an underestimate of the ratio between bone marrow and the low uptake compartment. The former estimate was determined from SPECT/CT in 15 patients as the ratio between the activity concentration in L4 and the surrounding tissue (*23*). In this study, we were able to compare the true ratio between L4 and the low-uptake compartment and found an almost 2 times higher ratio. Using this factor will result in a comparable median absorbed dose between the planar and the L4-SPECT methods in patients without metastasis, that is, 0.32 Gy/7.4 MBq.

The ability to measure the activity concentration in vertebrae for dosimetry is appealing; however, the low activity concentration in vertebrae is challenging because of the risk of cross contamination of scattered photons from surrounding high-uptake organs and tumors. To minimize this effect, we used the Monte Carlo–based reconstruction code SARec (22) for generating the SPECT images. This method reduces the influence of scattered photons and improves the recovery compared with ordered-subset expectation maximization reconstructions with and without recovery corrections (22). Nevertheless, the highest activity concentrations were observed in the vertebrae closest to the high-uptake organs. Studies using MRI demonstrated that the fraction between bone marrow and fat varies throughout the vertebral column and with sex and age (13–15). These studies found that the bone marrow fat fraction is larger in the lumbar vertebrae than in the thoracic vertebrae, consistent with our results of lower activity concentrations in the lumbar vertebrae. Furthermore, Baum et al. (15) reported that the bone marrow fat fraction (L1–L4) is 1.2 times higher in women than men in their sixties. Here, most patients were over 60 y old and men had a 1.13 times higher activity concentration; however, the difference was not statistically significant. Still, these results indicate that the variation in activity concentrations in the vertebrae might reflect the fat fraction and not cross contamination.

Because of the variable activity concentrations, we used different vertebrae to investigate the influence on the absorbed dose and the dose–response relationship. Previous studies mainly used L4 (26,30). When we used this single vertebra, we had to choose L5 or L3 in 15% of the patients because of skeletal metastases in L4. In contrast to the planar method, there was no dose–response correlation for patients without metastases and a rather strong correlation for patients with metastases using L4-SPECT. Similar results, with stronger correlations, were obtained when we used the median activity concentration in all, lumbar, or thoracic vertebrae. Thus, the hybrid methods might better reflect the influence of infiltrating bone marrow metastases on the absorbed dose. The absorbed doses were also higher in patients with metastases (except when using the planar method and T-SPECT), despite excluding metastases in the L4-SPECT method and using the median value to reduce the impact of metastases in the estimate. We demonstrated here that the platelet response is influenced by infiltrating metastases and previously that the absorbed dose to the spleen influences the platelet response by acting as a reservoir for platelets (31). In addition, age, sex, and pretreatment must be incorporated when modeling platelet response and developing predictive models for toxicity.

## CONCLUSION

The bone marrow absorbed doses differed between the methods studied and between patients with and without metastases. Nevertheless, image-based dosimetry methods demonstrated that increased absorbed doses result in higher platelet toxicity.

## DISCLOSURE

No potential conflict of interest relevant to this article was reported.

## KEY POINTS

**QUESTION:** Is the bone marrow absorbed dose and its correlation with hematologic response during ^177^Lu-DOTATATE treatments influenced by the image-based dosimetry method and the presence of skeletal metastases?
**PERTINENT FINDINGS:** In a cohort study of 46 patients treated with ^177^Lu-DOTATATE, it was established that a planar image–based method for bone marrow dosimetry was not influenced by skeletal metastases whereas hybrid dosimetry methods, based on planar and SPECT images, was influenced by skeletal metastases and by the vertebrae used for SPECT quantification. Nevertheless, all methods demonstrated statistically significant dose–response correlations; the planar method had the best correlation for patients without skeletal metastasis, and the hybrid methods had the best correlation for all patients and the cohort with skeletal metastasis.
**IMPLICATIONS FOR PATIENT CARE:** Different dosimetry methods might be required for individual prediction of hematologic toxicity in patients with and without skeletal metastases.
