# Supplementary material for: Bone Marrow Absorbed Doses and Correlations with Hematologic Response During 177Lu-DOTATATE Treatments Are Influenced by Image-Based Dosimetry Method and Presence of Skeletal Metastases
Source: J Nucl Med. 2019 Oct;60(10):1406–13. doi: 10.2967/jnumed.118.225235 (PMC6785794; doi:10.2967/jnumed.118.225235)
Supplement: Supplementary file 1 [file jnm225235SupplementalData.pdf]

Supplemental Table 1. Patient and dosimetry data for the first treatment cycle.

No.	Age	Gender	Skeletal metastases	Dosimetry method (Gy/7.4 Gy)					Relative platelet counts
				Planar	V-SPECT	L-SPECT	T-SPECT	L4-SPECT	
3	59	f	0	0.12	0.19	0,21	0,18	0,15	0,71
4	46	m	1	0.14	0.25	0,22	0,30	0,16	0,77
5	52	m	0	0.17	0.40	0,25	0,49	0,25	0,96
6	35	m	1	0.17	0.31	0,27	0,35	0,19	0,57
7	55	m	1	0.19	1.02	1,02	0,95	0,86	0,64
8	59	m	0	0.16	0.39	0,38	0,41	0,32	0,62
9	54	f	1	0.23	0.97	0,80	1,22	0,71	0,38
10	68	m	0	0.17	0.38	0,33	0,57	0,26	0,70
11	77	f	0	0.19	0.28	0,30	0,28	0,17	0,58
12	64	m	1	0.14	0.39	0,41	0,37	0,41	0,80
13	66	m	1	0.14	0.55	0,55	0,58	0,43	0,35
16	43	m	0	0.21	0.60	0,67	0,60	0,34	0,45
17	77	f	0	0.16	0.43	0,43	0,48	0,39	0,70
18	79	f	1	0.22	1.72	1,61	2,14	1,45	0,65
19	68	f	1	0.20	0.77	1,29	0,61	0,77	0,53
20	57	f	0	0.21	0.49	0,33	0,58	0,22	0,59
21	54	f	0	0.13	0.29	0,29	0,25	0,35	0,91
22	61	f	0	0.21	0.38	0,34	0,50	0,34	0,41
23	71	m	1	0.19	0.45	0,58	0,41	0,52	0,47
24	67	f	1	0.17	0.39	0,36	0,42	0,36	0,78
25	68	f	0	0.19	0.37	0,40	0,36	0,32	0,61
26	69	f	1	0.18	0.39	0,29	0,42	0,29	0,85
27	61	f	0	0.14	0.41	0,47	0,39	0,46	0,66
28	65	f	1	0.24	0.66	1,04	0,58	0,52	0,45
29	78	m	1	0.28	1.62	1,29	1,78	1,33	0,73
30	44	f	1	0.19	0.31	0,34	0,28	0,36	0,78
31	70	m	1	0.25	0.47	0,45	0,48	0,39	0,56
32	47	m	1	0.12	1.39	1,39	1,92	0,42	0,61
33	70	f	0	0.12	0.26	0,29	0,19	0,21	0,89
34	50	f	1	0.14	0.42	0,30	0,50	0,30	0,67
35	64	m	1	0.15	0.30	0,29	0,31	0,27	0,67
36	60	f	0	0.20	0.39	0,27	0,90	0,23	0,70
37	50	m	1	0.13	0.41	0,40	0,44	0,19	0,71
38	60	m	0	0.13	0.31	0,31	0,35	0,20	0,96
39	64	m	1	0.18	0.31	0,38	0,28	0,38	0,62
40	67	m	1	0.16	0.42	0,38	0,42	0,38	0,69
41	84	f	1	0.27	1.04	1,45	0,91	1,01	0,51
42	69	f	0	0.16	0.51	0,52	0,50	0,43	0,67
43	61	f	0	0.30	0.46	0,47	0,46	0,38	0,85
44	49	m	1	0.17	0.23	0,21	0,27	0,19	0,93
45	55	m	1	0.29	0.52	0,53	0,50	0,52	0,69
46	72	m	0	0.19	0.50	0,25	0,60	0,18	0,85
47	76	m	0	0.23	0.40	0,40	0,40	0,40	0,67
48	62	f	0	0.20	0.63	0,44	0,71	0,38	0,42
49	73	m	0	0.20	0.34	0,35	0,33	0,33	0,66
50	77	m	0	0.33	0.56	0,56	0,67	0,53	0,40

Supplemental Table 2. Patient and dosimetry data for the second treatment cycle.

No	Age	Gender	Skeletal metastases	Dosimetry method					Relative platelet counts
				Planar	L4-SPECT	V-SPECT	L-SPECT	T-SPECT	
3	59	f	0	0,12	0,24	0,21	0,24	0,24	0,77
4	46	m	1	0,14	0,25	0,23	0,23	0,23	0,68
5	52	m	0	0,17	0,29	0,49	0,36	0,36	0,81
6	35	m	1	0,17	0,39	0,41	0,68	0,68	0,16
7	55	m	1	0,19	0,47	0,72	0,65	0,65	0,47
8	59	m	0	0,16	0,28	0,30	0,30	0,30	0,62
9	54	f	1	0,23	0,42	0,65	0,56	0,56	0,63
10	68	m	0	0,17	0,25	0,35	0,28	0,28	0,66
11	77	f	0	0,19	0,21	0,23	0,22	0,22	0,73
12	64	m	1	0,14	0,20	0,24	0,21	0,21	0,67
13	66	m	1	0,14	0,19	0,37	0,22	0,22	0,34
16	43	m	0	0,21	0,45	0,60	0,59	0,59	0,36
17	77	f	0	0,16	0,43	0,46	0,43	0,43	0,72
18	79	f	1	0,22	0,92	0,93	0,92	0,92	0,61
19	68	f	1	0,20	0,70	0,70	0,74	0,74	0,38
20	57	f	0	0,21	0,21	0,39	0,32	0,32	0,64
21	54	f	0	0,13	0,31	0,29	0,27	0,27	0,77
22	61	f	0	0,21	0,34	0,34	0,30	0,30	0,29
23	71	m	1	0,19	0,26	0,46	0,40	0,40	0,37
24	67	f	1	0,17	0,35	0,39	0,36	0,36	0,64
25	68	f	0	0,19	0,31	0,43	0,43	0,43	0,47
26	69	f	1	0,18	0,28	0,33	0,33	0,33	0,80
27	61	f	0	0,14	0,25	0,29	0,25	0,25	0,69
28	65	f	1	0,24	0,65	0,66	0,93	0,93	0,28
29	78	m	1	0,28	0,62	1,01	0,83	0,83	0,48
30	44	f	1	0,19	0,23	0,28	0,24	0,24	0,69
31	70	m	1	0,25	0,40	0,49	0,49	0,49	0,58
32	47	m	1	0,12	0,55	1,04	1,04	1,04	0,53
33	70	f	0	0,12	0,17	0,42	0,68	0,68	0,90
34	50	f	1	0,14	0,36	0,37	0,36	0,36	0,62
35	64	m	1	0,15	0,36	0,28	0,36	0,36	0,64
36	60	f	0	0,20	0,29	0,39	0,29	0,29	0,74
37	50	m	1	0,13	0,17	0,31	0,29	0,29	0,89
38	60	m	0	0,13	0,24	0,32	0,26	0,26	0,96
39	64	m	1	0,18	0,30	0,24	0,30	0,30	0,89
40	67	m	1	0,16	0,29	0,29	0,29	0,29	0,65
41	84	f	1	0,27	0,71	0,65	0,71	0,71	0,15
42	69	f	0	0,16	0,70	0,55	0,60	0,60	0,77
43	61	f	0	0,30	0,70	0,54	0,60	0,60	0,59
44	49	m	1	0,17	0,22	0,27	0,25	0,25	0,90
45	55	m	1	0,29	0,46	0,46	0,46	0,46	0,53
46	72	m	0	0,19	0,23	0,50	0,33	0,33	0,70
47	76	m	0	0,23	0,53	0,53	0,53	0,53	0,57
48	62	f	0	0,20	0,34	0,47	0,40	0,40	0,29
49	73	m	0	0,20	0,35	0,40	0,40	0,40	0,55
50	77	m	0	0,33	0,40	0,50	0,50	0,50	0,34
